# Associations of circulating omentin-1 levels and long noncoding RNA MALAT1 expression with coronary heart disease in patients with type 2 diabetes mellitus

**DOI:** 10.1038/s41598-025-01153-5

**Published:** 2025-05-11

**Authors:** Meimei Tian, Jinchao Cao, Min Li, Pingping Lou, Huijie Ma, Yan Liu, Yukun Li

**Affiliations:** 1 Department of Endocrinology, The Third Hospital of Hebei Medical University, Ziqiang Road 139, Shijiazhuang City, 050051 Hebei Province China; 2 Department of Pediatric Orthopedics, The Third Hospital of Hebei Medical University, Ziqiang Road 139, Shijiazhuang City, 050051 Hebei Province China; 3 Department of Physiology, Hebei Medical University, Zhongshan East Road 361, Shijiazhuang City, Hebei Province China; 4 Hebei Collaborative Innovation Center for Cardio- Cerebrovascular Disease, Shijiazhuang, 050051 China; 5 Department of Endocrinology, The Third Hospital of Hebei Medical University, Ziqiang Road 139, Shijiazhuang City, 050051 Hebei Province China

**Keywords:** Type 2 diabetes mellitus, Coronary heart disease, Omentin-1; long noncoding RNA MALAT1, Biomarkers, Cardiology, Diseases, Endocrinology, Risk factors

## Abstract

Coronary heart disease (CHD) is a severe diabetic vascular complication and the main cause of mortality among diabetes patients. Early diagnosis of CHD could prevent its development. Both omentin-1 (Oment-1) and the long noncoding RNA MALAT1 (lncRNA MALAT1) can be detected in peripheral blood and exhibit protective or detrimental effects on CHD. However, whether these two factors could be predictive of CHD in T2DM patients remains unclear. Therefore, this study aimed to investigate the associations of circulating Oment-1 levels and the expression of MALAT1 with CHD in T2DM patients and to assess their predictive efficacy. A total of 137 T2DM patients were enrolled, including 68 patients without CHD (T2DM group) and 69 patients with CHD (T2DM + CHD group). Clinical parameters were collected, and plasma Oment-1 was measured by enzyme-linked immunosorbent assay (ELISA). RNA was isolated from peripheral monocytes, and the expression of MALAT1 was determined by quantitative PCR. Cardiac function was measured by echocardiography. Compared with that in T2DM patients, the plasma Oment-1 level was significantly lower, while the expression of MALAT1 was significantly greater in T2DM + CHD patients (all P values < 0.01). Bivariate correlation analysis indicated that Oment-1 was positively correlated with the left ventricular ejection fraction (LVEF) (*P* < 0.01). MALAT1 expression was negatively correlated with LVEF but positively correlated with age and DM duration (*P* < 0.05). Binary logistic regression suggested that Oment-1 and MALAT1 were significantly associated with the presence of CHD. Receiver operating characteristic (ROC) curve analysis demonstrated that both Oment-1 (AUC = 0.663, sensitivity = 75%, specificity = 49%) and MALAT1 (AUC = 0.749, sensitivity = 73%, specificity = 66%) had significant diagnostic value for CHD among T2DM patients. Notably, the combination of Oment-1 and MALAT1 exhibited better diagnostic efficiency (AUC = 0.771, sensitivity = 66.7%, specificity = 75.3%). In conclusion, decreased circulating Oment-1 levels and increased MALAT1 expression are closely associated with CHD in T2DM patients, and their combination offers superior diagnostic efficiency, suggesting Oment-1 and MALAT1 may serve as a non-invasive tool for the early CHD detection and risk stratification in high-risk T2DM patients. Further studies are warranted to explore the pathophysiological mechanisms of Omentin-1 and MALAT1 in the pathogenesis of CHD in T2DM and to validate their clinical utility as potential biomarkers in large cohort studies.

## Introduction

The global prevalence of diabetes mellitus (DM) has been increasing; the number of DM patients in China is 140.9 million, and this number is estimated to reach 174.4 million by 2045^1^. Coronary heart disease (CHD) is a macrovascular disease of T2DM and the major cause of cardiovascular mortality^2^. However, the parameters used in the clinic to evaluate cardiovascular diseases are more specific to severe cardiovascular events such as myocardial infarction, which cannot provide enough information for the risk of CHD in T2DM patients. Therefore, finding predictive biomarkers would be valuable for the early diagnosis and prevention of CHD in T2DM patients.

Omentin-1 (Oment-1) is a novel adipocytokine that has long been found to be protective against cardiovascular diseases^3^ and T2DM-associated comorbidities^4,5^ because of its anti-inflammatory and antioxidative effects^6,7^. Reduced circulating Oment-1 levels were found in CHD patients^8^ and is considered a cardiovascular risk biomarker in axial spondyloarthritis patients^9^ and in postmenopausal women^10^. However, few studies have evaluated the level and value of circulating Oment-1 in T2DM patients with CHD.

The role of lncRNAs in cardiovascular diseases has been recognized in recent years, and some lncRNAs could be used as biomarkers for the diagnosis of CHD^11^. The lncRNA metastasis-associated lung cancer transcript 1 (MALAT1) was originally found in lung cancer but is also one of the most studied lncRNAs in cardiovascular disease^12^. Elevated MALAT1 expression in peripheral blood was found in patients with coronary slow flow^13^, CHD patients^14^ and CHD patients with unstable angina^15^, indicating that MALAT1 has diagnostic value for CHD and related events. However, few studies have examined the role of MALAT1 in CHD among T2DM patients.

Atherosclerosis and inflammation are the primary pathological alterations in CHD. Intriguingly, Oment-1 is an anti-inflammatory and anti-atherogenic adipokine^16^, and circulating Omentin-1 levels are negatively correlated with atherosclerosis^17^. In contrast, MALAT1 has been shown to promote inflammation^18^, and its circulating expression is positively correlated with the progression of atherosclerosis^14^. However, the correlation between omentin-1 and MALAT1 has not yet been investigated.

Therefore, this study aimed to evaluate the roles of Oment-1 and MALAT1 in CHD among T2DM patients and to explore the potential roles of Oment-1, MALAT1 and their combination as specific noninvasive markers for CHD in T2DM patients.

## Materials and methods

### Patient recruitment

This study was conducted in the Department of Endocrinology, the Third Hospital of Hebei Medical University, from October 2021 to October 2023. A total of 137 T2DM patients aged 18–75 years were ultimately enrolled according to the inclusion and exclusion criteria. The inclusion criterion was as follows: T2DM diagnosed according to the WHO criteria (1999)^19^. The exclusion criteria were as follows: T2DM patients who were pregnant; presented with diabetes-related acute complications, such as diabetic ketoacidosis, hyperglycemic hyperosmolar state, and lactic acidosis; or had comorbidities, such as infectious diseases, autoimmune diseases, malignancies, heart failure, and renal and hepatic functional impairment.

CHD was diagnosed by meeting one of the following criteria^20^: at least one coronary artery stenosis > 50%, confirmed by previous coronary angiography or coronary CTA; a history of myocardial infarction; stable angina pectoris; and asymptomatic myocardial ischemia.

Patients were divided into two groups according to CHD history: the T2DM without CHD group (T2DM, *n* = 68) and the T2DM with CHD group (T2DM + CHD, *n* = 69). All patients with CHD received standard medical therapy, including aspirin and statins, and remained free from new episodes of myocardial infarction, angina pectoris, and heart failure.

This study was approved by the Ethics Committee of the Third Hospital of Hebei Medical University (Approval Code. W2021-088-1) and performed in accordance with relevant guidelines, and written informed consent was obtained from each participant. The study was performed in accordance with the Declaration of Helsinki.

## Patient data and biochemical parameter collection

The baseline data of the patients, including age, sex, height, weight, duration of diabetes, family history, smoking status and alcohol consumption, were recorded. The abdominal circumference, height and weight were measured, and BMI was calculated as body weight (kg)/height (m)2 (kg/m2).

Blood samples were collected from the patients after 8 h of overnight fasting. The levels of triglycerides (TGs), total cholesterol (TC), low-density lipoprotein cholesterol (LDL-C), high-density lipoprotein cholesterol (HDL-C), very low-density lipoprotein cholesterol (VLDL-C), uric acid (UA), creatinine (Cr), alanine aminotransferase (ALT), aspartate aminotransferase (AST), C-reactive protein (CRP), and homocysteine (Hcy) were measured via photoelectric colorimetry. Hemoglobin A1c (HbA1c) was measured via high-performance liquid chromatography. Fasting C-peptide (FC-P) and 25-hydroxy vitamin D (25(OH)D) were measured via MAGLUMI^@ ^Chemiluminescence Immunoassy System(Snibe Co.,Ltd.Shenzhen) and available kits(cat.no.130205001M, cat.no.130261004M,  Snibe Co.,Ltd.Shenzhen). The estimated glomerular filtration rate (eGFR) was calculated using the Chronic Kidney Disease Epidemiology Collaboration equation^21^.

## Echocardiography

Transthoracic echocardiography was carried out using a color ultrasound diagnostic instrument (PHILIPS EPIQ7 C). The thickness of the ventricular wall and left ventricular end-diastolic diameter (LVEDD) were measured by M-mode ultrasound. The method of LVESD measurement was the same as that of LVEDD, and the left ventricular ejection fraction (LVEF) was calculated via the following formula: LVEF = (LVEDD- LVESD)/LVEDD.

## Oment-1 measurement

Fasting blood (5 ml) was collected from each patient, and plasma was collected after centrifugation. The plasma samples were stored at − 80 °C before every measurement. The plasma level of Oment-1 was measured by enzyme-linked immunosorbent assay (ELISA) by commercial kits (CUSABIO, Wuhan; Catalog No. CSB-E09745 h) according to the manufacturer’s protocol.

## RNA isolation and RT‒qPCR

Fasting blood samples were collected, and peripheral monocytes were isolated by commercially available kits (Beijing Solabor Technology Co., Ltd.). RNA was extracted via the TRIzol method (cat. no. 15596026; Invitrogen; Thermo Fisher Scientific, Inc.), and RNA quality and concentration were assessed by UV spectroscopy at 260 and 280 nm. A HiFiScript gDNA Removal cDNA Synthesis Kit (cat. no. CW2582S; CoWin Biosciences) was used for reverse transcription. MonAmp ChemoHS qPCR mix (cat. no. rn04005M; Monad Biotech Co., Ltd.) was used to detect MALAT1 expression, and β-actin was used as a housekeeping gene. The thermocycling conditions were as follows: 95 °C for 15 min; 40 cycles at 95 °C for 10 s, 56 °C for 30 s and 72 °C for 30 s. Relative gene expression was calculated via the 2^−△△CT^ method.

The PCR primers were designed and synthesized by Sangon Biotech Co., Ltd. (Shanghai). The sequences of primers used were as follows:

β-Actin: forward primer, 5’-AAGGCCAACCGCGAGAA-3’; reverse primer, 5’-ATGGGGGAGGGCATACC-3’. LncRNA MALAT1: forward primer 5’-TACCTAACCAGGCATAACA-3’; reverse primer 5’-GTAGACCAACTAAGCGAAT-3’.

### Statistical analysis

All the statistical analyses were performed using SPSS software (version 26). The normality of the continuous data was examined via the Shapiro–Wilk test. Normally distributed data are expressed as the means ± standard deviations (means ± SDs), whereas nonnormally distributed data are expressed as medians and interquartile ranges (IQRs). Independent sample t tests or Mann‒Whitney U tests were performed to assess the differences between two groups. Categorical variables are expressed as numbers and were compared using the chi-square test. Spearman’s rank correlation analysis and Pearson’s correlation analysis were performed to evaluate the correlations between the study factors and the clinical and biochemical parameters. Binary logistic regression was performed to determine the contributions of the study parameters to the prediction of CHD onset. Receiver operating characteristic (ROC) curve analysis was performed to evaluate the diagnostic value of parameters for CHD in T2DM patients. A two-sided P value < 0.05 was considered statistically significant.

## Results

### Demographic, clinical, and biochemical characteristics of the study subjects

In the present study, there were no significant differences in patients’ baseline characteristics between T2DM patients and T2DM + CHD patients, including sex, BMI, family history, smoking status, or alcohol consumption, (all *P* > 0.05). Compared with T2DM patients without CHD, the duration of diabetes was significantly longer in T2DM patients with CHD (*P* = 0.019).

Compared with those in T2DM patients, SBP, HbA1c and FBG were significantly greater in T2DM + CHD patients (all *P* < 0.05), whereas LVEF, TC, LDL-C and eGFR were significantly lower in T2DM + CHD patients (all *P* < 0.05). There were no differences in DBP or other biochemical parameters, including TG, HDL-C, VLDL-C, ALT, AST, Cr, UA, CRP, FC-P, Hcy and 25(OH)d, between the two groups (all *P* > 0.05) (Table 1).

**Table 1:** Demographic, clinical and biochemical characteristics of the study subjects.

Characteristic	T2DM	T2DM + CHD	*P* value
Number	68	69	
Age (year)	52.05 ± 12.04	65.17 ± 11.42	0.001^*^
Gender (female/male)	32/36	34/35	0.193
DM Duration (years) Abdominal circumferences(cm)	8.03 ± 6.80 92.76 ± 8.10	15.95 ± 6.61 92.96 ± 8.70	0.019^*^ 0.894
BMI(kg/m^2^)	25.89 ± 3.27	25.31 ± 3.23	0.306
HbA1c (%)	7.76 ± 1.65	8.73 ± 1.94	0.002^*^
DM Family history (yes/no)	26/42	29/40	0.688
Smoking (yes/no)	29/39	20/49	0.095
Drinking alcohol (yes/no)	24/44	21/48	0.275
SBP(mmHg)	130.16 ± 16.41	138.41 ± 17.81	0.006^*^
DBP(mmHg)	86.57 ± 13.45	86.22 ± 10.96	0.865
FBG(mmol/L)	7.75 ± 2.62	10.14 ± 3.84	< 0.001*
TC (mmol/L)	5.17 ± 1.36	4.61 ± 1.21	0.011^*^
TG (mmol/L)	2.62 ± 1.88	1.66 ± 1.20	0.051
LDL-C (mmol/L)	3.01 ± 0.76	2.69 ± 0.82	0.015^*^
HDL-C (mmol/L)	1.20 ± 0.27	1.19 ± 0.25	0.264
VLDL-C (mmol/L)	1.19 ± 1.76	0.75 ± 0.54	0.052
ALT (U/L)	31.34 ± 27.94	28.45 ± 26.76	0.538
AST (U/L)	22.90 ± 14.59	22.78 ± 11.51	0.959
Cr (µmol/L)	70.04 ± 31.87	69.92 ± 21.03	0.656
eGFR(ml/min/1.73 m^2^)	99.59 ± 22.06	88.56 ± 18.48	0.002^*^
UA (µmol/L)	334.07 ± 103.04	314.25 ± 90.37	0.102
CRP (mg/L)	4.83 ± 1.13	3.69 ± 1.33	0.537
FC-P (ng/mL)	2.64 ± 1.13	2.38 ± 1.18	0.239
Hcy(µmol/L)	12.38 ± 4.65	12.88 ± 4.86	0.850
25(OH)D(ng/ml)	27.74 ± 6.76	16.25 ± 5.22	0.165
LVEF(%)	56.41 ± 4.42	50.63 ± 3.35	< 0.001*

BMI: body mass index; HbA1c: hemoglobin A1c; SBP: systolic pressure; DBP: diastolic blood pressure; FBG: fasting blood glucose; TC: total cholesterol; TG: triglycerides; HDL-C: high-density lipoprotein cholesterol; LDL-C: low-density lipoprotein cholesterol; VLDL-C: very low-density lipoprotein cholesterol; ALT: alanine aminotransferase; AST: aspartate aminotransferase; Cr: creatinine; eGFR: estimated glomerular filtration rate; UA: uric acid; CRP: C-reactive protein; FC-P: fasting C peptide; Hcy: homocysteine; 25 (OH) D: 25-hydroxyvitamin D; LVEF: left ventricular ejection fraction.

* *p* < 0.05, T2DM + CHD vs. T2DM.

### Oment-1 levels and MALAT1 expression in circulation

**Fig. 1:**
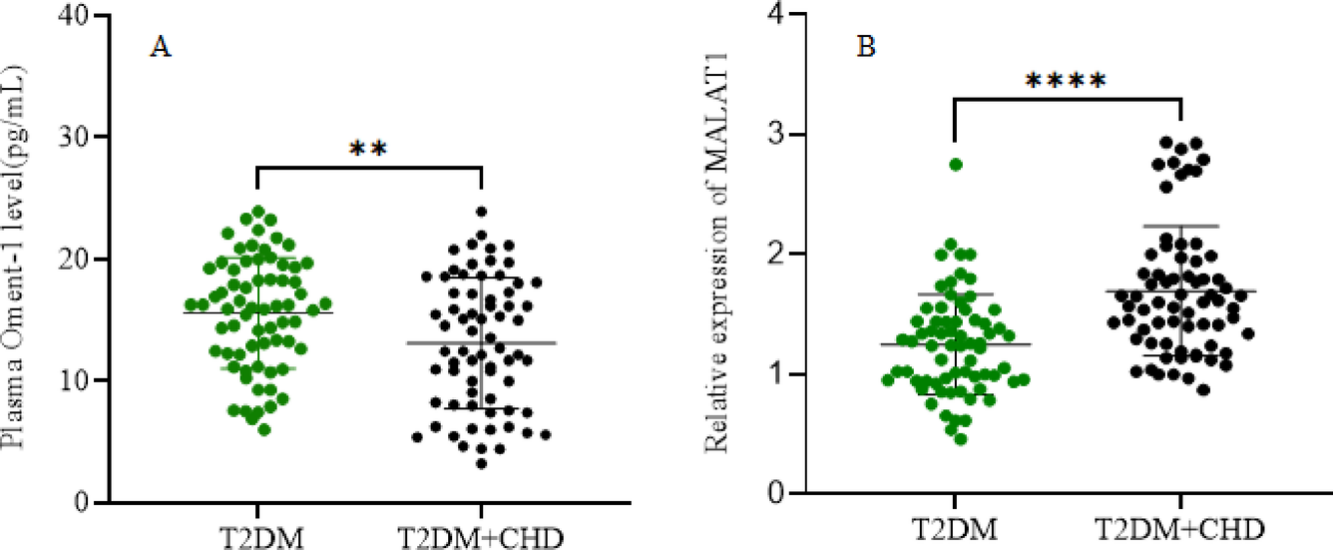
Oment-1 levels and MALAT1 expression in the circulation of T2DM and T2DM + CHD patients. Compared with that in T2DM patients, the level of Oment-1 was significantly decreased (A), whereas the expression of MALAT1 (B) was significantly increased in the circulation of T2DM + CHD patients. * *p* < 0.05 for T2DM + CHD vs. T2DM.

In the present study, compared to T2DM patients, plasma Oment-1 levels were significantly decreased in T2DM + CHD patients (15.55 ± 4.55 pg/ml vs. 13.09 ± 5.35 pg/ml, *P* = 0.0043; Fig. 1A), whereas MALAT1 expression in peripheral blood cells was significantly increased in T2DM + CHD patients (1.25 ± 0.42 vs. 1.69 ± 0.54, *P* < 0.0001; Fig. 1B). These findings indicated that reduced plasma Oment-1 levels and increased MALAT1 expression might be involved in the pathogenesis of CHD in T2DM patients.

### Correlation analysis of Oment-1 and MALAT1

Bivariate correlation analysis was performed for Oment-1 levels and MALAT1 expression to determine the relevant factors. Oment-1 was positively correlated with LVEF (*r* = 0.223, *p* = 0.001) and had no correlation with other parameters, including age, BMI, HbA1c, FBG, CRP, TC, TG, HDL-C, LDL-c, or eGFR. The expression of MALAT1 was negatively correlated with LVEF (*r* =−0.253, *p* = 0.007) but positively correlated with age (*r* = 0.451, *p* = 0.002) and DM duration (*r* = 0.201, *p* = 0.019) (Table 2).

**Fig. 2:**
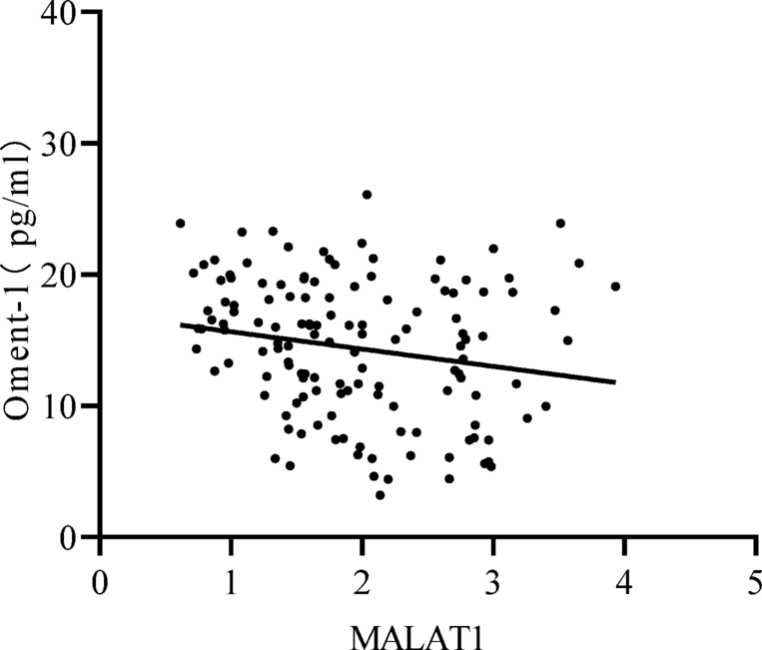
Correlation of Oment-1 levels with MALAT1 expression in patients with T2DM. The Oment-1 level was negatively correlated with MALAT1 expression (*r* = −0.19, *p* = 0.026).

We also investigated the correlation between Oment-1 and MALAT1 and found that the level of plasma Oment-1 was negatively correlated with MALAT1 expression (*r* = −0.19, *p* = 0.026) (Fig. 2).

**Table 2:** Correlation analysis of Circulating Oment-1 and MALAT1.

	Oment-1		MALAT1	
	r	P	r	P
Age(year)	−0.023	0.794	0.451	0.002*
DM Duration(year)	−0.016	0.849	0.201	0.019*
BMI	−0.008	0.926	−0.085	0.322
HbA1c(%)	−0.078	0.378	−0.041	0.643
FBG(mmol/L)	0.028	0.748	−0.079	0.361
CRP (mg/L)	0.169	0.132	0.063	0.574
TC(mmol/L)	−0.060	0.487	0.004	0.963
TG(mmol/L)	−0.056	0.520	−0.057	0.514
HDL-C(mmol/L)	0.125	0.150	−0.032	0.709
LDL-C(mmol/L)	−0.014	0.876	0.050	0.563
VLDL-C(mmol/L)	−0.055	0.523	−0.057	0.513
UA(µmol/L)	−0.008	0.931	−0.103	0.235
eGFR(ml/min/1.73 m^2^)	0.134	0.121	−0.057	0.513
FC-P(ng/mL)	−0.005	0.954	−0.167	0.062
Hcy(µmol/L)	−0.091	0.334	−0.061	0.519
LVEF(%)	0.223	0.001*	−0.253	0.007*

The data are presented as correlation coefficients (r).

Abbreviations are listed in Table 1.

*Statistically significant (*p* < 0.05).

### Association of Oment-1 and MALAT1 with the presence of CHD

Binary logistic regression analysis was performed with the presence of CHD in T2DM patients as the dependent variable and the study variables (Oment-1, MALAT1) as independent predictors. The regression models and data are shown in Table 3. In the first regression model, only the study variables were taken as predictors, and both Oment-1 and MALAT1 were significantly associated with the presence of CHD. In the second model, after adjusting for age, sex, duration of diabetes and HbA1c, Oment-1 and MALAT1 were still significantly associated with the presence of CHD. These associations were still significant even after adjusting for age, sex, duration of diabetes, HbA1c, BMI, LDL-C and eGFR, as shown in the third model (Table 3).

**Table 3:** Binary logistic regression analysis of the independent factors for the presence of CHD (Oment-1 and MALAT1 as continuous variables).

Model	OR	95% CI	*P* value
1			
Oment-1	0.891	0.8423–0.965	0.004^*^
MALAT1	8.886	3.330–23.590	< 0.001^*^
2			
Oment-1	0.856	0.776–0.945	0.002^*^
MALAT1	7.104	2.363–21.361	< 0.001^*^
Duration (years)	1.017	0.958–1.080	0.575
Age	1.088	1.034–1.144	0.001*
Gender	0.912	0.323–2.591	0.863
HbA1c	0.752	0.564–1.004	0.053
3			
Oment-1	0.853	0.750–0.970	0.015^*^
MALAT1	4.147	1.217–14.133	0.023^*^
Duration (years)	1.040	0.966–1.121	0.300
Age	1.098	1.102–1.192	0.024*
Gender	1.016	0.266–3.883	0.981
HbA1c	0.774	0.513–1.170	0.224
BMI	1.076	0.895–1.294	0.437
LDL-C	0.833	0.398–1.745	0.629
eGFR	1.016	0.982–1.051	0.353
LVEF	0.564	0.442–0.754	< 0.001^*^

Model 1: not adjusted for any variable; Model 2: adjusted for age, sex, duration of diabetes and HbA1c; Model 3: adjusted for Model 2, BMI, LDL-C and eGFR. OR: odds ratio; 95% CI: 95% confidence interval.

*Statistically significant (*p* < 0.05).

### ROC curve analysis

**Fig. 3:**
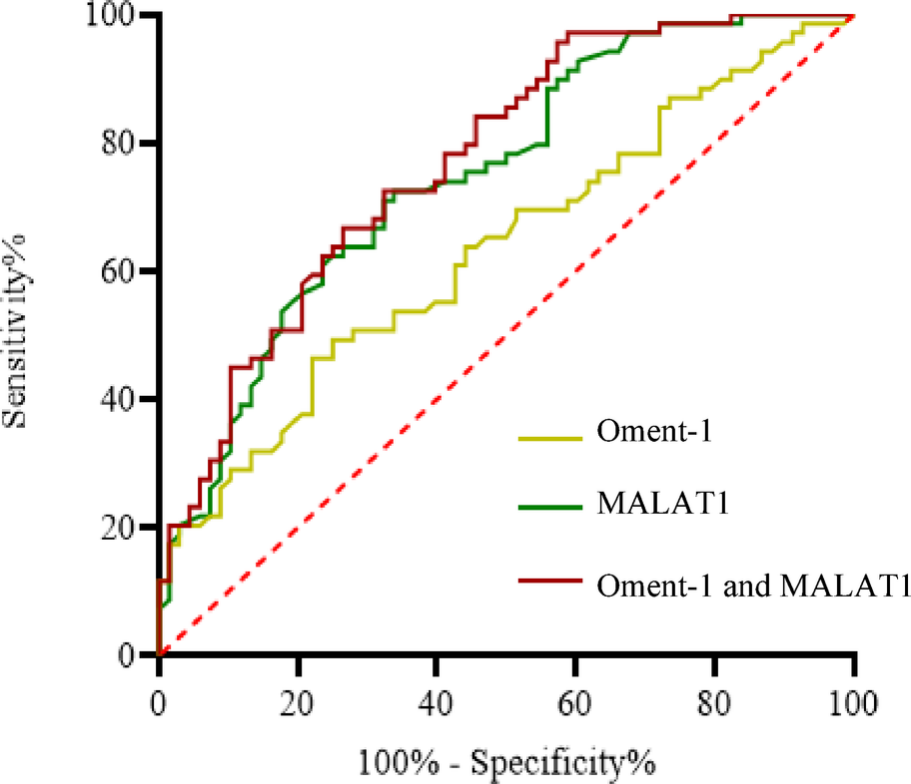
Oment-1, lncMALAT1 and their combination to predict T2DM with CHD. ROC curves indicated that both Oment-1 and MALAT1 had significant predictive value for T2DM patients with CHD, and the predictive value was even greater when these two factors were combined.

ROC curve analysis was performed to determine the diagnostic value of Oment-1 and MALAT1 for the presence of CHD in T2DM patients. On the basis of analyses of the ROC curves (Fig. 1), plasma Oment-1 levels with cutoff values ≤ 12.45 pg/ml were used to discriminate CHD patients from non-CHD patients with T2DM, with an AUC of 0.633 (95% CI: 0.540–0.725; *p* < 0.001), a sensitivity of 75%, a specificity of 49%, a PPV of 59.9%, and an NPV of 65.9%. The cutoff value of MALAT1 expression was ≥ 1.367, with an AUC was 0.749 (95% CI: 0.668–0.830; *p* < 0.001), a sensitivity of 73%, a specificity of 66%, a PPV of 68.6%, and an NPV of 70.7%. Additionally, the AUC of Oment-1 combined with MALAT1 was 0.771 (95% CI: 0.694–0.848; *p* < 0.001), the sensitivity was 66.7%, the specificity was 75.3%, the PPV was 73.3%, and the NPV was 69.1%, indicating that the combination of Oment-1 and MALAT1 had better diagnostic value for CHD in T2DM patients. (Fig. 3)

## Discussion

T2DM patients are at a greater risk of developing cardiovascular disease, which is the leading cause of mortality among this population^22^. In the present study, we investigated the correlations between circulating Oment-1 levels and MALAT1 expression in T2DM patients with CHD. Data have indicated that, compared with those in T2DM patients without CHD, circulating Oment-1 levels are significantly lower, whereas MALAT1 expression is increased in CHD patients. Additionally, the plasma Oment-1 level was negatively correlated with LVEF, whereas MALAT1 expression was positively correlated with age, DM duration and LVEF. Notably, Oment-1 and MALAT1 were inversely correlated with each other. Both factors, either independently or in combination, exhibited predictive value for CHD in T2DM patients.

In the present study, we found that the level of circulating Oment-1 was lower in T2DM patients with CHD and that it has predictive value for CHD. Similar results have also been reported previously in CHD patients with diabetes^23^ or without diabetes^24,25^. The favorable effect of Oment-1 on CHD might be due to its endothelial cell protective and anti-atherosclerotic effects. Oment-1 has been found to partly ameliorate FFA-induced endothelial cell injury^26^ and improve endothelial function by activating the Akt/eNOS/NO^27^ and AMPK/PPARδ pathways^28^, contributing to increased NO production and the inhibition of ER stress and oxidative stress. Oment-1 can modulate macrophage function and inhibit atherosclerosis formation and development^29–31^.

Intriguingly, this was the first study to find that circulating Oment-1 levels were positively correlated with LVEF. Similar results have been reported in patients with dilated cardiomyopathy^32^ and in mouse models of heart failure^33^, indicating that Oment-1 levels might also be related to ventricular diastolic function. In vivo studies have shown that Oment-1 can prevent pathological cardiac remodeling following ischemia^34^ and ameliorate ischemia-induced myocardial injury by activating mitophagy and maintaining dynamic mitochondrial homeostasis^35^. An in vitro study indicated that Oment-1 could protect H9 C2 cells from docetaxel-induced damage by reducing endoplasmic reticulum stress^36^. Therefore, Oment-1 is a protective factors against cardiomyocytes, and its effect on diabetic cardiomyopathy is a prospective area that needs to be further investigated.

In this study, we detected the expression of the lncRNA MALAT1 in peripheral blood mononuclear cells from T2DM patients and found that the expression of MALAT1 in the circulation was increased in T2DM patients with CHD. Similar results have also been reported by Sohrabifar et al.^37^ in Iranian patients. Elevated expression of MALAT1 in the circulation has been reported in CHD patients^38,39^ and is associated with increased CHD severity^38^ and unstable angina^40^, indicating that MALAT1 could be a biomarker for CHD screening and surveillance.

In the present study, we found that MALAT1 expression in the circulation of T2DM patients was positively correlated with age and diabetes duration. No similar results have been reported previously in T2DM patients. A previous study performed in patients with periodontitis and healthy subjects revealed that blood MALAT1 expression was not correlated with age^41^. The data from the present study suggest that MALAT1 might be more susceptible to the influences of aging and duration in T2DM patients. MALAT1 is known to promote inflammation and oxidative stress. Thus, the elevated MALAT1 levels in older patients with longer durations of diabetes might reflect cumulative inflammatory damage, potentially exacerbating cardiovascular complications.

Additionally, this was the first study to find that MALAT1 expression was negatively correlated with LVEF in patients with T2DM. A previous study performed by Qi et al.^42^ reported that, in hemodialysis patients with heart failure, lncRNA ENST00000561762 expression in peripheral blood mononuclear cells was negatively correlated with LVEF. Therefore, MALAT1 expression in peripheral blood might also play a crucial role in the diagnosis of cardiac function in T2DM patients. Some evidence has indicated that MALAT1 contributes to high glucose-induced cardiomyocyte damage by causing mitochondrial damage and oxidative stress^43^. It remains unclear whether MALAT1 expression in peripheral blood mononuclear cells reflects MALAT1 expression in heart tissue.

To the best of our knowledge, this is the first study to investigate the correlation between circulating Oment-1 levels and MALAT1 expression in patients with T2DM. Our results revealed a significant negative correlation between Oment-1 and MALAT1. Given that Oment-1 has anti-inflammatory and antioxidative effects, whereas MALAT1 is associated with proinflammatory activity, our findings suggest a potential regulatory network involving adipokines (e.g., Oment-1) and lncRNAs (e.g., MALAT1) in the pathogenesis of diabetic vascular complications. However, further studies are needed to elucidate the underlying mechanisms and clinical implications.

This study has several limitations. First, it is a single-center study with a relatively small sample size, which may cause patient selection bias. Second, the use of hypoglycemic or lipid-lowering drugs could influence circulating Oment-1 levels and MALAT1 expression. Additionally, since T2DM patients with CHD are older than those without CHD are, age-related factors might also affect Oment-1 and MALAT1 levels. Third, serum 25(OH)D levels could be influenced by vitamin D supplementation; therefore, its role requires further investigation. Future studies with larger sample sizes are needed to better understand the clinical implications of these findings.

## Conclusions

Decreased circulating Oment-1 levels and elevated MALAT1 expression are significantly associated with CHD in patients with T2DM, suggesting their potential as biomarkers for the noninvasive early detection of CHD in T2DM patients. However, further validation through large-scale, multicenter studies is needed to confirm their diagnostic efficacy and determine clinically relevant cutoff values.

## Data Availability

Data are available on request from the corresponding author.
